# Evaluation of the efficacy of a cannabidiol and cannabidiolic acid rich hemp extract for pain in dogs following a tibial plateau leveling osteotomy

**DOI:** 10.3389/fvets.2022.1036056

**Published:** 2023-01-04

**Authors:** Sarah Klatzkow, Garrett Davis, Justin Shmalberg, Aitor Gallastegui, Erin Miscioscia, Jason Tarricone, Lindsay Elam, Matthew D. Johnson, Katelyn M. Leonard, Joseph J. Wakshlag

**Affiliations:** ^1^Department of Small Animal Surgery, Red Bank Veterinary Hospital, Tinton Falls, NJ, United States; ^2^Department of Small Animal Clinical Sciences, College of Veterinary Medicine, University of Florida, Gainesville, FL, United States; ^3^Department of Clinical Sciences, Cornell College of Veterinary Medicine, Ithaca, NY, United States

**Keywords:** cannabidiol, CBD, cannabidiolic acid, hemp, canine

## Abstract

**Objective:**

To determine the impact of a cannabidiol (CBD) and cannabidiolic acid (CBDA) rich hemp product on acute post-operative pain in dogs following a tibial plateau leveling osteotomy (TPLO), and to evaluate for changes in early bone healing, serum chemistry profiles, and complete blood counts.

**Methods:**

In this randomized, placebo controlled, blinded clinical trial, 44 client-owned dogs were assigned to receive either a CBD/CBDA product dosed at 2–2.5 mg/kg PO every 12 h or a placebo for 4 weeks following a TPLO. Variables evaluated before (week 0), and at 2 and 4 weeks post-operatively included standardized veterinary assessments for pain score, weight-bearing, and lameness, the Canine Brief Pain Inventory (pain interference score–PIS, pain severity score–PSS), and serum biochemistry. Complete blood counts were performed at weeks 0 and 4. Additionally, orthogonal radiographs evaluating the degree of healing were taken at week 4. A mixed model analysis, analyzing changes of variables of interest from enrollment baseline to all other time points was utilized, with a *p*-value ≤ 0.05 considered significant.

**Results:**

Of the 44 enrolled patients, 3 were lost to follow up and excluded from analysis. No significant differences were noted between placebo (*n* = 19) and CBD/CBDA (*n* = 22) groups at any point in pain score, degree of lameness, degree of weight-bearing, PIS, PSS, or radiographic healing of the osteotomy. A significant finding of elevation of ALP above normal reference range in the treatment group was identified (*p* = 0.02) and eosinophil count was affected by treatment (*p* = 0.01), increasing from baseline in placebo and decreasing in treatment groups. Finally, a significant difference (*p* = 0.03) was noted at 2 weeks post-operatively where 4 patients in the placebo group and no treatment patients received trazodone to facilitate activity restrictions.

**Clinical significance:**

Use of a CBD/CBDA rich hemp product dosed at 2–2.5 mg/kg PO every 12 h did not have a significant impact on pain or delay early bone healing. A statistically significant increase in ALP, decrease in eosinophils, and reduced use of trazodone was identified in the treatment group.

## Introduction

Cannabinoids have been used therapeutically in human medicine for a variety of ailments including epilepsy, anxiety, depression, sleep disorders, nausea, glaucoma, Multiple Sclerosis, Parkinson's, Alzheimer's, and chronic or neuropathic pain (1). Use of cannabinoids has quickly gained traction in veterinary medicine, fueled by its myriad of uses in human medicine and changes in federal and state regulations resulting in the legal sale and use of these products (2, 3).

Endocannabinoids occur naturally in mammals, maintaining homeostasis by acting on cannabinoid receptors throughout the body, involving neuronal pathways and potentially the immune system to help modulate pain and inflammation. The 2 main receptors of this endocannabinoid system are cannabinoid receptor 1 (CB1) and cannabinoid receptor 2 (CB2). CB1 is primarily located within the central nervous system, and plays a role in neuropathic pain modulation, movement, and memory processing. CB2 is predominantly located within cells related to the immune function such as B-cells and natural killer cells, where it can modulate the inflammatory response. This role is complex and characterized by release of anti-inflammatory cytokines combined with inhibition of pro-inflammatory mediators, through inhibition of inflammatory cell migration and T cell proliferation, and modulation of the production and signaling of cytokines and chemokines, including TNF-α, IFN-γ, IL-6, and IL-10 (4–11). CB2 receptors have been identified in murine and human bones, with the endocannabinoid system playing a role in regulation of bony mass and remodeling (12). Phytocannabinoids are plant produced cannabinoids which influence the same receptors in mammals and are the therapeutic basis of cannabinoid rich hemp products.

Cannabis sativa L. is the strain of hemp plant from which therapeutic cannabinoids are derived. The plant contains over 100 cannabinoids, with the most abundant being cannabidiolic acid (CBDA) and tetrahydrocannabinolic acid (THCA). Better known and marketed compounds are cannabidiol (CBD) and tetrahydrocannabinol (THC) which are the decarboxylated form of the prior molecules produced during heat extraction when processing hemp products (13–18). THC is responsible for the psychotropic activity in cannabis primarily through interactions at the CB1 receptor in the central nervous system, while CBD, CBDA and THCA have no psychotropic effects and are widely regarded as being highly tolerable with minimal reported adverse side effects (5, 7, 8, 14, 15, 19–22).

More importantly, since CBD, CBDA and THCA are not known agonists of the CB1 and are weak agonists of the CB2 receptors at very high concentrations, much of the mechanism for nociception appears to be through other receptors involved in pain perception that are considered part of the endocannabinoid system and their interaction with the endogenous ligands anandamide and 2-arachidonyoylglycerol. At the level of CB receptors, CBD can inhibit the reuptake of anandamide, resulting in increased levels. The interaction of CBD with these receptor systems includes modulation of the transient receptor potential channels (TRPV1) through increase of endogenous ligand anandamide, agonist for glycine receptor activity, serotonin release through direct and indirect activation of the 5-hydroxytryptophan receptor (5HT1A) by CBD and anandamide, and direct agonist of CBD on peroxisomal proliferation activation receptor gamma (PPAR -γ) (4, 5, 7, 15, 23).

Despite the increasing popularity, there remains limited literature on the use of CBD rich hemp-based products in veterinary medicine, with the current knowledge of the full extent of the impact on pain being limited, particularly as it relates to acute surgical pain (11). Disagreement exists among reports regarding the efficacy of CBD for use in veterinary patients, with multiple studies reporting benefits in chronic osteoarthritis pain and epilepsy (6, 9, 24, 25) and a single report suggesting no significant improvement in pain (26). To date, there has been no formal study on the use of CBD in post-operative patients. The primary objective of this study was to determine the impact of a CBD/CBDA-rich hemp product on acute post-operative pain in dogs associated with a tibial plateau leveling osteotomy (TPLO) when administered for 4 weeks post-operatively. The hypothesis was that a CBD/CBDA-rich hemp product would reduce acute pain scores as compared to a placebo control. Secondary objectives were to evaluate radiographs at week 4 post-operatively to assess for changes in early bone healing and to examine serum chemistry profiles and complete blood count for changes throughout the study period.

## Materials and methods

Forty-four dogs were enrolled from a large private practice specialty hospital and veterinary university hospital from August 2019 to January 2021 in a randomized, placebo-controlled, blinded clinical trial. Enrollment was based on a power analysis (UBC Power/Sample Size Calculator, β- 0.80, α-0.05) expecting a reduction of approximately 20 points on the Canine Brief Pain Inventory with a standard deviation of approximately 20 points based on Gamble et al. (9) resulting on a need for minimally 16 dogs per group. It was assumed that there could be dropouts due to the nature of the clinical trial; hence the goal for enrollment was 22 per group, allowing for a 25% dropout rate. Patients were eligible for inclusion in the study if there was a diagnosed unilateral cranial cruciate ligament rupture with no significant concurrent orthopedic, neurologic, or systemic disease. A tibial plateau leveling osteotomy (TPLO) was recommended as the treatment of choice and surgery was performed by a board-certified surgical specialist. All owners were informed of the study and consented to have their dogs enrolled. With the exception of non-steroidal anti-inflammatory drugs, medications and supplements outside the study design were discouraged for at least 2 weeks prior to the surgery, and for 4 weeks following surgery. All patients were treated with a NSAID for 5 days following surgery. Each owner agreed to have their dog evaluated at time zero, 2 weeks and 4 weeks following surgery. Owners were informed that throughout the study period, the use of new medications, supplements, or dose changes should be minimized and reported to investigators and may result in exclusion from the study. Throughout the study, all assigned capsules, bloodwork, radiographs, and sedation performed at the designated time points were provided at no cost to the owner. No direct compensation or waived fees were provided for the surgery, medications, or visits outside the scope of this trial.

All dogs were anesthetized and received intra-operative and immediate post-operative pain control including injectable opiates and ultrasound guided nerve blocks at the discretion of the attending anesthesiologist and surgeons managing the case. All TPLOs were unilateral and performed by a single board-certified surgeon at each facility using standard accepted surgical techniques. Partial medial meniscectomy was performed in all cases of meniscal tears and recorded. Dogs were hospitalized overnight and were treated for immediate post-operative pain at the discretion of the attending surgeon. The day following surgery, all dogs were initiated on a 5-day course of a NSAID and a 28-day course of either CBD/CBDA-rich hemp oil or placebo capsules. Carprofen was administered in all but 3 patients (all in the treatment group) who pre-operatively were receiving meloxicam, firocoxib, or grapiprant. The use of antibiotics was at the discretion of the treating veterinarian. Use of sedatives such as trazodone was discouraged by clinicians and only prescribed if requested by the owner to help minimize the dog's physical activity. Use of trazodone or other sedatives was recorded. All patients were discharged from hospital the day following surgery.

Each dog was randomly assigned into the CBD/CBDA treatment or placebo group using a random number generator (Randomizer iPhone application) for a total of 22 dogs in each group. The treatment consisted of a hydrocarbon extracted, hemp derived cannabinoid product emulsified in sesame seed oil (ElleVet Sciences, Portland, ME, USA) from a United States Department of Agriculture hemp facility that is certified and audited annually for good manufacturing practices in compliance with the 2018 Farm Bill. The oil suspension was utilized to make 10 mg, 25 mg, and 50 mg CBD/CBDA containing capsules to be dispensed. A certificate of analysis of the batch of product used in this study was performed by an ISO 17025 accredited third-party laboratory (ProVerde Laboratories, Milford MA, USA) and was approximately 30 mg/mL of CBD, 31 mg/mL of CBDA, 1.2 mg/mL THC, 1.3 mg/mL THCA, 1 mg/mL of cannabichromene and 1.2 mg/mL of cannabichromenic acid. The certification of the hemp product passed all quality control measures regarding microbial, mycotoxin, pesticide, heavy metal and solvent contamination. The placebo was formulated utilizing the same volume of sesame seed oil in similar capsules. Patients were dosed with variations in numbers of CBD/CBDA capsules at 2–2.5 mg/kg body weight orally every 12 h. Containers holding the capsules were labeled as A or B, based on placebo or treatment, to keep owners and clinicians blind to treatment.

Time zero was defined as a point no > 2 weeks prior to a scheduled surgery date. At this evaluation, clients were educated as to what was involved with the study, including the potential that their dogs may be placed in a placebo group, and provided informed consent to participate in the study under an approved IACUC from the University of Florida and compliance with institutional guidelines. Patients presented to one of 2 sites (Red Bank Veterinary Hospital, Red Bank NJ [RB]; University of Florida Veterinary Hospitals, Gainesville, FL [FL]). An initial client survey asked owners to report any travel plans or guests in the home anticipated during the study period, quantify the number of episodes of vomiting, diarrhea, and lethargy over the previous 2 weeks, and to document dosages of any medications or supplements the patient had received over the previous 2 weeks.

**Figure 1:**
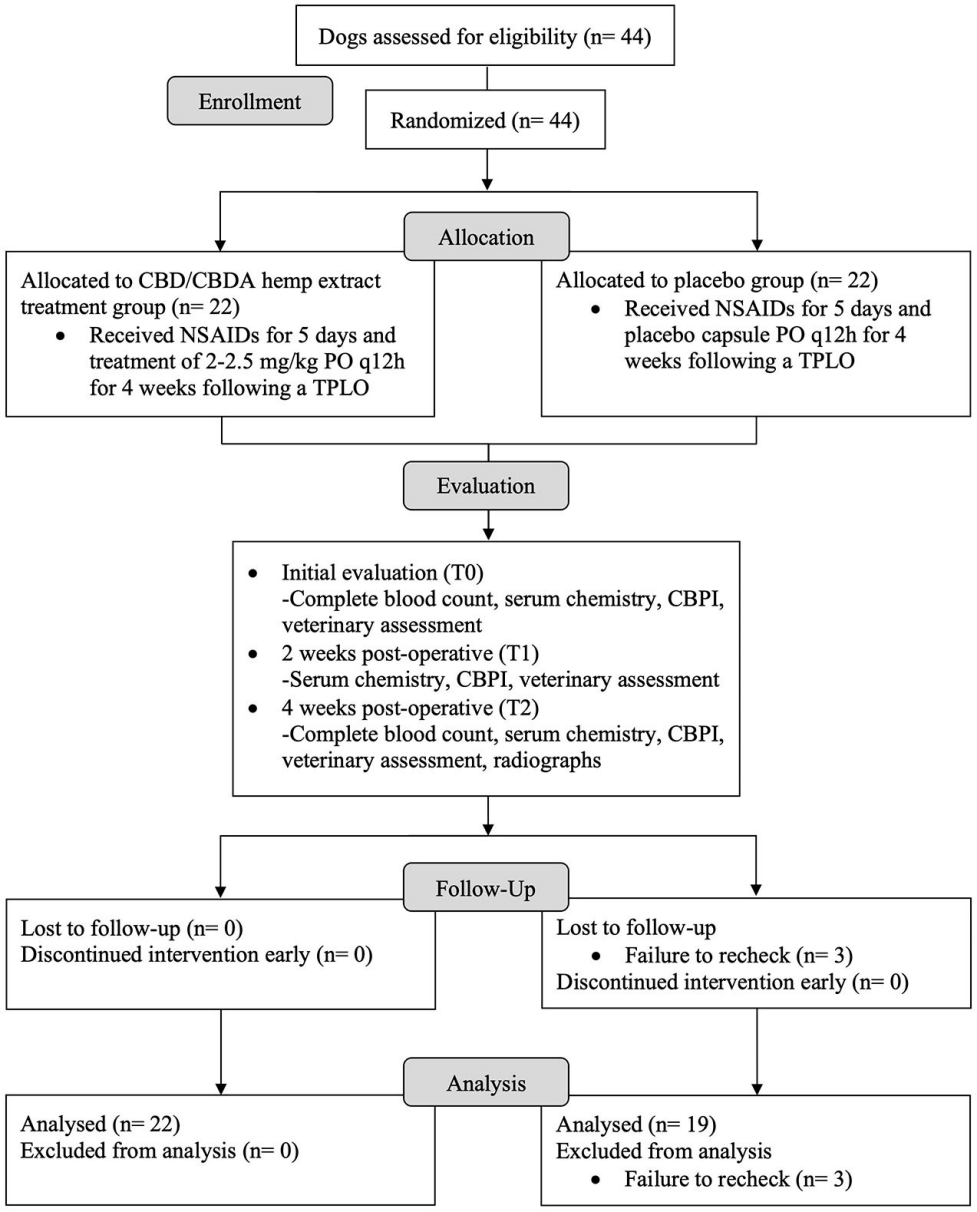
Flow diagram for a randomized, blinded, placebo-controlled design study to evaluate the effects of a cannabidiol (CBD) and cannabidiolic acid (CBDA) rich hemp product on post-operative pain, complete blood count, serum biochemistry, and early bone healing in dogs following a tibial plateau leveling osteotomy (TPLO).

At initial evaluation (T0 or week 0), 2 weeks (+/– 2 days) post-operative (T1), and 4 weeks (+/– 2 days) post-operative (T2), all patients were examined by a single participating investigator at each hospital. At each evaluation, patients underwent a standardized veterinary assessment performed by either investigator which evaluated patient lameness, pain, and weight-bearing on scales of 1-5 based on standard descriptors as summarized in Table 1. Each dog had a complete blood count performed at T0 and T2, and chemistry performed at all 3 time points. Bloodwork was performed at either Antech Diagnostics or the University of Florida Veterinary Hospital's Diagnostic Clinical Pathology Laboratory. At each time point, the owners completed a Canine Brief Pain Inventory (CBPI). For the purpose of this study, the overall quality of life index was not considered as dogs were expected to score better after surgical intervention regardless of group. Additionally, at T2, standardized orthogonal TPLO radiographs, including a 90/90 flexed lateral stifle and a craniocaudal stifle projections, were obtained. Sedation could be performed as needed to facilitate the acquisition of well positioned radiographs at the safety of patients and staff. A schematic of the study design is provided in Figure 1.

**Table 1:** Standardized veterinary assessment scoring system used to evaluate all dogs in the study, before and after a tibial plateau leveling osteotomy (TPLO).

**Criterion**	**Grade**	**Clinical evaluation**
Lameness	1	Walks normally
	2	Slightly lame when walking
	3	Moderately lame when walking
	4	Severely lame when walking
	5	Reluctant to rise, recumbent
Pain	1	No pain elicited
	2	Mild, turns head
	3	Moderate, resists and vocalizes
	4	Severe, growls and shows teeth
	5	Will not allow manipulation
Weight-bearing	1	Equal on all limbs standing and walking
	2	Normal standing, favors limb when walking
	3	Partially weight-bearing walking and standing
	4	Partial weight-bearing walking, sits immediately when not walking
	5	Non-weight-bearing at stand or walk

Patients were evaluated at time intervals of T0 (initial evaluation at week 0), T1 (2 weeks post-operatively), and T2 (4 weeks post-operatively). Pain was evaluated on both palpation and range of motion of the affected stifle.

All radiographs from T2 were evaluated by a single board-certified radiologist who was blinded to group assignments. Radiographs were evaluated for the degree of callus formation, distinctness of the osteotomy gap line, stage of union, and assigned a healing score based on a system proposed by Hammer et al. (27).

### Statistical analysis

Statistical analysis was performed with a commercially available software package (JMP 10.0; Cary NC, USA). Demographics (sex, age, body condition score [BCS] and body weight in kilograms) were assessed across the treatment groups using Student's *t*-test (age, BCS and weight) or Fisher's exact testing (sex) to assess group differences. Veterinary assessment scores (pain, lameness, and weight-bearing) and CBPI were assessed using a Wilcoxon Signed rank test to compare placebo and treatment groups. All data were assessed utilizing a Shapiro-Wilks test for normality and residual plots were examined to determine normality, and when normality was rejected the data was log transformed and visually inspected for normal distribution before analysis utilizing a two-way analysis of variance with repeated measures for serum chemistry, and without repeated measures for complete blood count assessments. Variables included in the model were: fixed effects of treatment, time, treatment x time. Tukey's tests were performed *post hoc* on any significant effects of time, or time x treatment to assess differences found. Due to different clinic sites, a similar mixed model was utilized for CBPI and veterinary scores (pain, lameness, gait) with the added variable of trial site (FL or RB). Bone healing parameters were assessed using a Wilcoxon Signed rank test to compare bone healing between placebo and treatment groups for the 4 week follow up radiographs. As NSAIDs (pain relief) and trazodone (sedative) were used in some patients at baseline and continued throughout the trial period, a Fisher's exact test was performed between groups at T1 and T2 to assess whether there were differences in use of these drugs at these time points compared to baseline. A *p*-value of < 0.05 was determined to be significant for all analyses.

## Results

Forty-one patients met the inclusion criteria and completed the study of the 44 originally enrolled. Of the dogs completing the study, 15 dogs were male; 2 were intact and 13 neutered. Twenty-six were female and all were spayed. The median age of patients was 7 years (range 1–13 years, mean 6.5 years). Median weight of patients was 31 kg (range 20.3–53.4 kg, mean 33.3 kg). Median body condition score graded on a scale of 1–9 was 6/9 (range 4-9/9, mean 6.05/9). During the trial 3 patients in the placebo group were lost to follow up leaving only 19 dogs in the placebo group with 22 dogs in the treatment group that completed the trial. No significant differences were noted between the 2 groups.

### Veterinary assessments

The veterinary assessment evaluated a patient's lameness, pain score, and degree of weight-bearing on a scale of 1–5 at all 3 time points. Improvement was determined as a decrease in score on each of these scales.

The median and range of scores are summarized (Table 2). For lameness assessment there was an effect of time across all time points regardless of treatment group (*P* < 0.001). No treatment or treatment over time effect was found. Similarly, pain scores were found to decrease over the time of the trial (*P* < 0.001) with no effect of treatment or treatment over time. Finally, for degree of weight-bearing, effect of time was also found to be significant (*P* < 0.01); however, an effect of treatment or treatment over time could not be found. In addition, pain scoring appeared to differ across the 2 sites used in this study with RB pain scoring suggesting lower pain scores compared to FL (*P* < 0.001).

**Table 2:** Results of lameness, pain, and weight-bearing scores graded on a 1–5 scale (1 = best; 5 = worst) in dogs treated with either a cannabidiol (CBD) and cannabidiolic (CBDA) rich hemp product (2–2.5 mg/kg orally every 12 h; *n* = 22) or a placebo (sesame seed oil every 12 h, *n* = 19) for 4 weeks after a tibial plateau leveling osteotomy surgery.

**Score**	**Placebo T0**	**Placebo T1**	**Placebo T2**	**Tx T0**	**Tx T1**	**Tx T2**	**P site**	**P_Tx_**	**P_Time_**	P_Tx*time_
Lameness	3 (1–4)	2.5 (2–3)	2 (1–3)	3 (1–4)	3 (2–4)	2 (1–3)	0.92	0.25	<0.001	0.98
Pain	3 (1–3)	1 (1–3)	1 (1–3)	3 (2–3)	2 (1–2)	1 (1–3)	<0.001	0.91	<0.001	0.66
Weight-bearing	3 (1–4)	3 (2–3)	2 (1–3)	3 (1–5)	3 (2–3)	2.5 (1–3)	0.28	0.17	<0.001	0.70

Results reported as median (range) at week 0 (T0; initial evaluation before surgery) and at 2 and 4 weeks post-operatively (T1 and T2, respectively). *P*-values < 0.05 considered significant and evaluated effect of site (FL vs. RB) treatment (P_Tx_), effect of time (P_Time_), and effect of treatment over time (P_Tx*time_) by a mixed model analysis of Wilcoxon Signed rank test and Tukey's tests.

### Canine brief pain inventory

The Canine Brief Pain Inventory was categorized as pain severity score (PSS) and pain interference score (PIS and measured at all 3 time points. Mean and standard deviation are summarized (Table 3). For the PSS and PIS there was significant effect of time observed (*P* < 0.001); however, a treatment effect or treatment over time effect were not observed.

**Table 3:** Results of pain severity score (PSS) and pain interference scores (PIS) in dogs treated with either a cannabidiol (CBD) and cannabidiolic (CBDA) rich hemp product (2–2.5 mg/kg orally every 12 h; *n* = 22) or a placebo (sesame seed oil every 12 h, *n* = 19) for 4 weeks after a tibial plateau leveling osteotomy surgery.

**Score**	**Placebo T0**	**Placebo T1**	**Placebo T2**	**Tx T0**	**Tx T1**	**Tx T2**	**P site**	**P_Tx_**	**P_Time_**	P_Tx*time_
PSS	19 ± 7	12 ± 6	6 ± 6	20 ± 9	12 ± 8	6 ± 6	0.37	0.88	<0.001	0.77
PIS	30 ± 12	21 ± 12	10 ± 8	38 ± 15	23 ± 15	11 ± 11	0.78	0.24	<0.001	0.29

Results reported as mean ± SD at week 0 (T0; initial evaluation before surgery) and at 2 and 4 weeks post-operatively (T1 and T2, respectively). *P*-values < 0.05 considered significant and evaluated effect of site (FL vs. RB) treatment (P_Tx_), effect of time (P_Time_), and effect of treatment over time (P_Tx*time_) by a mixed model analysis of Wilcoxon Signed rank test and Tukey's tests.

### Clinical pathology

All clinicopathologic findings were summarized (Table 4). Complete blood counts assessed at the beginning and end of the trial showed that only eosinophil counts were affected by treatment (*P* = 0.01), as eosinophil counts increased in the placebo group and decreased in the treatment group. A treatment over time effect was not observed. Serum chemistry data was normally distributed for all parameters except for serum ALT and serum ALP, which were log transformed before analysis. Serum chemistry evaluations across the entire cohort revealed an increase in potassium (*P* < 0.01), a decrease in glucose (*P* < 0.02), a decrease in ALT (*P* = 0.03), and a decrease in AST (*P* = 0.05) from baseline regardless of treatment over time. For ALP, an effect of treatment was noted whereby the treatment group showed rises above reference ranges for the respective lab regardless of time, while the placebo group showed decreases in ALP from baseline over time (*P* = 0.02).

**Table 4:** Clinicopathologic values of complete blood count and serum biochemistry in dogs treated with either a cannabidiol (CBD) and cannabidiolic (CBDA) rich hemp product (2–2.5 mg/kg orally every 12 h; *n* = 22) or a placebo (sesame seed oil every 12 h, *n* = 19) for 4 weeks after a tibial plateau leveling osteotomy surgery.

**Variable**	**Placebo T0**	**Placebo T1**	**Placebo T2**	**Tx T0**	**Tx T1**	**Tx T2**	**P_Tx_**	**P_Time_**	P_Tx*time_
Total Protein (g/dL)	6.4 ± 0.7	6.4 ± 0.6	6.4 ± 0.6	6.2 ± 0.5	6.4 ± 0.5	6.4 ± 0.5	0.58	0.61	0.45
Albumin (g/dL)	3.5 ± 0.4	3.4 ± 0.4	3.4 ± 0.4	3.3 ± 0.4	3.3 ± 0.3	3.3 ± 0.4	0.22	0.11	0.83
Globulin (g/dL)	2.9 ± 0.5	2.9 ± 0.5	2.9 ± 0.4	2.9 ± 0.3	3.1 ± 0.4	3.1 ± 0.4	0.59	0.13	0.41
AST (U/L)	35 ± 15	30 ± 13	28 ± 7	35 ± 30	28 ± 8	25 ± 6	0.72	0.05	0.41
ALT (U/L)	59 ± 49	43 ± 40	51 ± 50	45 ± 43	34 ± 27	36 ± 23	0.23	0.03	0.06
ALP (U/L)	101 ± 292	88 ± 178	70 ± 113	71 ± 129	192 ± 345	253 ± 477	0.02	0.03	0.46
T bili. (mg/dL)	0.2 ± 0.1	0.2 ± 0.1	0.2 ± 0.1	0.2 ± 0.1	0.2 ± 0.2	0.2 ± 0.1	0.44	0.18	0.61
BUN (mg/dL)	16 ± 4	18 ± 5	17 ± 4	14.0 ± 4	16 ± 4	16 ± 5	0.28	0.21	0.88
Creatinine (mg/dL)	1.0 ± 0.2	1.1 ± 0.2	1.1 ± 0.1	1.0 ± 0.2	1.0 ± 0.2	1.1 ± 0.2	0.64	0.16	0.88
Phosphorus (mg/dL)	3.8 ± 1.0	4.0 ± 0.7	3.7 ± 0.8	3.7 ± 1.1	3.8 ± 0.9	3.5 ± 0.9	0.75	0.13	0.84
Glucose (mg/dL)	99 ± 19	97 ± 14	97 ± 10	97 ± 25	86 ± 25	92 ± 26	0.89	0.02	0.19
Calcium (mg/dL)	10.0 ± 0.5	10.0 ± 3.3	10.0 ± 0.4	9.8 ± 0.6	10.0 ± 3.8	9.9 ± 0.4	0.29	0.06	0.58
Sodium (mEq/L)	147 ± 3	146 ± 2	147 ± 2	147 ± 3	147 ± 2	146 ± 3	0.95	0.44	0.02
Potassium (mEq/L)	4.4 ± 0.2	4.5 ± 0.3	4.5 ± 0.4	4.3 ± 0.3	4.5 ± 0.4	4.5 ± 0.6	0.77	<0.01	0.8
Chloride (mEq/L)	112 ± 3	119 ± 32	112 ± 2	113 ± 4	111 ± 4	112 ± 5	0.27	0.06	0.8
Cholesterol (mg/dL)	247 ± 91	261 ± 101	247 ± 87	271 ± 110	298 ± 116	288 ± 90	0.32	0.12	0.67
WBC (thous/uL)	9.81 ± 4.87	–	8.53 ± 2.09	9.72 ± 5.13	–	8.20 ± 2.27	0.85	0.08	0.89
RBC (mill/uL)	7.21 ± 0.87	–	7.42 ± 0.61	7.05 ± 0.91	–	7.32 ± 0.62	0.67	0.04	0.76
HGB (g/dL)	17.0 ± 2.0	–	17.5 ± 1.5	16.9 ± 2.1	–	17.6 ± 1.51	0.83	0.02	0.65
Hct (%)	50.1 ± 5.8	–	50.9 ± 4.4	49.8 ± 5.9	–	51.6 ± 4.9	0.82	0.1	0.49
Platelet (thous/uL)	281 ± 82	–	300 ± 89	265 ± 96	–	315 ± 97	0.08	0.01	0.03
Neutrophil (abs)	7240 ± 4612	–	5833 ± 4213	7167 ± 4913	–	5598 ± 1752	0.6	0.06	0.91
Lymphocyte (abs)	1743 ± 657	–	1848 ± 660	1505 ± 537	–	1691 ± 624	0.51	0.09	0.63
Monocyte (abs)	422 ± 285	–	473 ± 348	404 ± 216	–	370 ± 179	0.26	0.86	0.43
Eosinophils (abs)	381 ± 265	–	391 ± 238	619 ± 603	–	556 ± 323	0.01	0.67	0.56

Results reported as mean ± SD at week 0 (T0; initial evaluation before surgery) and at 2 and 4 weeks post-operatively (T1 and T2, respectively). Serum biochemistry was performed at all timepoints while complete blood count at T0 and T2. P-values < 0.05 considered significant and evaluated effect of treatment (P_Tx_), effect of time (P_Time_), and effect of treatment over time (${\text{P}}_{\text{T}{\text{x}}^{*}time}$) by a mixed model two–way analysis of variance and Tukey's test.

AST, aspartate animotransferase; ALT, alanine animotranferase; ALP, alkaline phosphatase; T bili, total bilirubin; BUN, blood urea nitrogen; WBC, white blood cells; RBC, red blood cell; HGB, hemoglobin; Hct, hematocrit; abs, absolute.

### Radiographic assessment

Radiographs were submitted for the 41 patients and reviewed by a board-certified radiologist for assessment of callus formation and healing scores according to methods described (28). There was a median callus formation score of 1 (range 0-3) in the placebo and median score of 1 (range 0–4) in the treatment group. There was a median healing score of 1 (range 0-3) in the placebo and 1 (range 0–4) in the treatment group. There were no significant differences between the degree of callus formation (*P* = 0.67) and subjective healing scores (*P* = 0.53) for either group.

### Additional medications

Carprofen remained the NSAID of choice for this study; however, 3 of the treatment patients received alternate NSAIDs as they historically were administered them, 1 each of meloxicam, firocoxib, and grapiprant. No difference was found between these patients and the remainder of the treatment group based on Fisher's exact test. Additionally, despite being prescribed for a 5 day course, owners continued to administer NSAIDs in a group of patients based on discretion and report of pain. At T1, there were 4 placebo and 5 treatment patients, while at T2 there were 1 placebo and 2 treatment patients still receiving NSIADs. There was no difference in pain scores identified between groups at either timepoint. Finally, although use of trazodone or other sedative was discouraged, there were 8 placebo and 9 treatment patients receiving trazodone at the time of surgery. At T1, 4 placebo and 0 treatment patients remained on trazodone, the difference was found to be significant on a Fisher's exact test (*P* = 0.03).

## Discussion

There is paucity information on the use of cannabinoid-rich hemp products to control post-operative pain. This study was conducted to determine the clinical effect of a CBD/CBDA-rich hemp product on acute pain in canine patients treated for a cranial cruciate ligament rupture with a routine, commonly practice TPLO surgical procedure with established outcomes. Clinical metrology instruments (veterinary assessments and CBPI) were used to assess pain for up to 4 weeks post-operatively. In this study, it was found that the only difference between groups were veterinary assessed pain scores between sites over time, leading the authors to reject the initial hypothesis that a CBD/CBDA rich hemp product would reduce acute post-operative pain scores compared to the control in this cohort.

Currently in the veterinary literature, clinical effects in osteoarthritis and refractory seizures suggests that a dose of approximately 2–2.5 mg/kg every 12 to 24 h shows clinical efficacy which were the tenants of dosing in our study (6, 8, 9, 15, 19, 26, 28, 29). While no benefit was identified in this present study, interestingly a recent abstract using the same product used in this study at a 5 mg/kg dose every 8 h may decrease pain scores in patients undergoing intervertebral disk disease surgery (30). Currently, no toxic dose has been established with reports of 20 mg/kg orally every 12 h for 6 weeks, 4 mg/kg once daily for 6 months, and lower doses of 2 mg/kg every 12 h for 12 weeks being tolerated in dogs (19, 28, 31).

CBDA is less well studied yet recent studies show that both CBDA and THCA demonstrate superior absorption in dogs than CBD. While the understanding of CBDA pharmacology remains lacking, CBDA has been shown to increase serum CBD concentrations with lower CBD dosing due to improved absorption and retention of CBD and CBDA (16, 17, 32). This process has been described as an “entourage effect,” by which CBDA and THCA work synergistically with CBD, lowering the dose of CBD/CBDA-rich hemp product required to meet similar therapeutic levels when compared to a purified CBD product (16). It may have been useful to assess serum steady state concentrations at the 4-week visit to better understand the effects of serum concentrations; however, at the time of study initiation, no commercial laboratories were assessing CBDA serum concentrations.

In this cohort, only two significant clinicopathologic changes were noted. The only treatment effects observed was a serum chemistry elevation in ALP. The elevation of liver values in this cohort, particularly ALP, is consistent with previous reports in human, murine, and canine literature, and thought to be due to induction of cytochrome p450 mediated oxidative metabolism (9, 14, 15, 19, 24–26). In a recent trial in dogs receiving 4 mg/kg daily of CBD for 6 months, similar rises in ALP were identified which returned to baseline within 4 weeks of cessation of CBD (31). However, elevation of liver enzymes is inconsistent in the literature, with multiple reports in canine patients having no identified changes, particularly in young healthy cohorts (6, 8, 15).

Additionally, there was a slight increase in eosinophils in the placebo group and a mild decrease identified in our treatment group, though still within reference ranges. Human eosinophils exposed *in vitro* to high concentrations of THC or CBD respond with increased expression of macrophage inflammatory protein-1β (MIP-1β), suggesting that THC or CBD may exacerbate pre-existing eosinophilic inflammatory disease (33). Studies in dogs have mixed results, suggesting mild anti-inflammatory effect when used at very high concentrations, with a more recent report in which oral CBD at typical dosing had no influence on immune cell regulation (34). To the authors knowledge, a relative eosinopenia associated with CBD use has not been reported in the literature and further studies are necessary to understand the significance particularly in light of the rise observed in the placebo group.

The most interesting finding in this study was the use of trazodone to limit activity and mildly sedate dogs throughout the study period. The use of sedative was discouraged and implemented based on owner insistence for the safety of the patient and implants. At the initial evaluation, 8 dogs in the placebo group and 9 dogs in the CBD/CBDA received trazodone. At the second visit at 2 weeks post-operatively, 4 placebo dogs remained on trazodone while 0 required trazodone in the treatment group, suggesting a potentially sedating effect of CBD/CBDA. In human and veterinary studies adverse effects of somnolence and lethargy have been noted with using CBD-rich hemp extracts (35, 36). Direct comparison of a CBD-rich hemp extract in a treat format given as approximately 0.7 mg/kg orally every 12 h was assessed in dogs identified with noise phobias showing that treatment with CBD did not impact behavioral anxiety scoring, while trazodone was mildly effective for some behavioral parameters (37). However, our dosing was significantly higher and contained a mix of CBD/CBDA rich hemp. Though not the primary outcome and a small sample size, these data suggest future study of CBD rich hemp products for agitation and anxiety are warranted in the post-surgical period for activity restriction.

CBD has been shown to increase the recruitment of mesenchymal stem cells and subsequent differentiation to osteoblastic lineage in experimental models (12). Additionally, CBD enhances mechanical properties of callus formation through expression of procollagen-lysine 2-oxoglutarate 5-dioxygenase, a collagen cross linking enzyme. When administered in murine studies, radiographic evidence of CBD stimulated callus formation was seen after week 6 (12, 38, 39). In the present study, no impact on bone healing from the treatment was observed.

Overall, this study had several limitations. While a control population was used, a confounding placebo effect or regression of the mean cannot be ruled out. A small population size was investigated in this study, though this is thought to have had minimal impact on the results as the sample size was determined by a power analysis. As this was multi-institutional, 2 principal examiners were involved and despite using a standardized veterinary assessment scoring system, pain scores differed between the 2 sites. Additionally, given that 2 clinical pathology laboratories were used, values were evaluated individually and in terms of the respective lab reference ranges, rather than being combined as means. An additional limitation is that a complete blood count was only performed at 2 time points as it was not anticipated to see change in eosinophil based on prior literature. While the use of additional medications (NSAIDs after 5 days or trazodone) was discouraged, owner insistence on the use remained an unavoidable factor. While prolonged use of NSAIDs or use of NSAIDs other than carprofen had no impact on results, there was a significant difference between groups with long term trazodone administration. Stricter exclusion criteria in regard to medications may be considered in future studies. Finally, there was a lack of standardization between anesthetic protocols which included the use of local nerve blocks. While this may have impacted immediate post-operative pain, it is not suspected to have a significant effect given an overall lack of efficacy of CBD/CBDA in the study.

In conclusion, the results of the current study indicate that when administered at a dose of 2–2.5 mg/kg twice daily for 4 weeks following a TPLO, the CBD/CBDA hemp extract had no effect on measures of pain or early bone healing. Administration was associated with an increase in ALP and a relative eosinopenia compared to a relative eosinophilia in the placebo group. Finally, there was a possible association of CBD/CBDA and reduced post-operative anxiety. Further investigation is warranted for this use, although possible negative effects on ALP and eosinophils should be considered.

## Data availability statement

The raw data supporting the conclusions of this article will be made available by the authors, without undue reservation.

## Ethics statement

The animal study was reviewed and approved by University of Florida IACUC and in compliance with Red Bank Veterinary Hospital Institutional Guidelines. Written informed consent was obtained from the owners for the participation of their animals in this study.

## Author contributions

SK was responsible for data interpretation, drafting of the manuscript revision, and approval of the submitted manuscript. GD, JS, EM, LE, and MJ were responsible for acquisition of data and manuscript revision. AG was responsible for interpretation, assessment of radiographic images, and manuscript revision. JT was responsible for acquisition of data, data entry, and manuscript revision. KL was responsible for data entry and manuscript revision. JW was responsible for the conception of the study, statistical analysis, and manuscript revision. All authors contributed to the article and approved the submitted version.
